# Non-Affinity Purification of Antibodies

**DOI:** 10.3390/antib12010015

**Published:** 2023-02-13

**Authors:** Tsutomu Arakawa, Yui Tomioka, Masataka Nakagawa, Chiaki Sakuma, Yasunori Kurosawa, Daisuke Ejima, Kouhei Tsumoto, Teruo Akuta

**Affiliations:** 1 Alliance Protein Laboratories, San Diego, CA 92130, USA; 2 Research and Development Division, Kyokuto Pharmaceutical Industrial Co., Ltd., Tahahagi 318-0004, Japan; 3 Bio-Diagnostic Reagent Technology Center, Sysmex Corporation, Sayama 350-1332, Japan; 4 Department of Bioengineering, School of Engineering, The University of Tokyo, Tokyo 113-8656, Japan

**Keywords:** non-affinity, purification, antibody, mixed-mode, MEP, agarose, salting-out, activated carbon, Protein A

## Abstract

Currently, purification of antibodies is mainly carried out using a platform technology composed primarily of Protein A chromatography as a capture step, regardless of the scale. However, Protein A chromatography has a number of drawbacks, which are summarized in this review. As an alternative, we propose a simple small-scale purification protocol without Protein A that uses novel agarose native gel electrophoresis and protein extraction. For large-scale antibody purification, we suggest mixed-mode chromatography that can in part mimic the properties of Protein A resin, focusing on 4-Mercapto-ethyl-pyridine (MEP) column chromatography.

## 1. Introduction

Protein A chromatography constitutes a purification platform technology, although expensive, for research and the commercial-scale production of antibodies. The process of antibody production at both small and large scales has been comprehensively reviewed [1,2]. In the research setting, the major problem with Protein A chromatography is not the high cost of Protein A resin but the difficulty in elution due to strong protein–protein interactions between antibodies and the Protein A ligand [3], which requires strong acid for reasonable recovery. This acid elution can cause denaturation and hence a loss of potency [4,5,6]. In addition, such a harsh elution condition can cause the leaching of Protein A, which can impact in vivo effects of the eluted antibodies [7,8]. Here, we review methods of antibody purification that do not use a Protein A column.

We previously showed that agarose native gel electrophoresis can separate antibodies and host cell contaminants effectively [9,10,11], and hence here investigated the feasibility of antibody purification using native gel electrophoresis. With this method, antibody binding, which may potentially cause protein denaturation or aggregation, is not involved. At the commercial scale, the cost of Protein A is a major issue, as low pH elution can cause damage not only to the antibodies to be purified but also the Protein A ligand, which can reduce the lifetime of the resin. Thus, the cost of using Protein A depends on how many cycles the ligand can be reused [12]. Other problems include the low binding capacity of Protein A resin and Protein A leaching [12]. To address these issues, attempts have been made to replace expensive Protein A resin with conventional chromatographic resins. Mixed-mode resins that combine hydrophobic properties with electrostatic properties for binding or elution have shown promising results. Namely, the mixed-mode resins have at least part of the Protein A properties in terms of their multiple binding mechanisms. They differ from single-mode resins such as ion-exchange and hydrophobic resins but lack complementary contact surfaces between Protein A and the antibody Fc structure. We here review several mixed-mode chromatography methods. In particular, the MEP (4-Mercapto-ethyl-pyridine) HyperCel resin, one of the mixed-mode chromatography, is designed to bind antibodies at physiological pH and elute the bound antibodies at mildly acidic pH [13,14]. Because of its simple process and our familiarity, we focus on the MEP chromatography.

## 2. Problems Associated with Protein A Chromatography

### 2.1. Cost

Protein A resin is expensive and labile to harsh denaturing treatments [12]. When a crude mixture of samples is loaded, it is recommended to sanitize and regenerate the column for subsequent reuse. Thus, the cost of the Protein A resin is determined by how many cycles the resin can be reused. It has been demonstrated that pre-treatments of the cell culture harvest, such as the preferential precipitation of antibodies [12] and preferential precipitation of host cell contaminants (e.g., lipids and chromatin) using allantoin, increased the lifetime of the Protein A resin [15,16,17]. For regeneration, most of the Protein A resins are resistant to the denaturing conditions of guanidine hydrochloride, urea, low pH and reducing agents. However, loss of the Protein A ligand occurs at each cycle of application due to leaching, ligand degradation and proteins that are irreversibly bound to the ligand [12].

### 2.2. Low pH Elution

It is known that antibodies can denature at low pH [4,5,6]. Acid-treated antibodies showed greater thermal stability of the CH2 domain and higher affinity for complement C1q binding [4]. The conformation and thermal stability of antibodies gradually change with decreasing pH, which results in irreversible damages to the pH-neutralized samples, including more oligomer species in antibody samples [2,3,18]. In addition, such low pH-induced structure changes have been attributed to the polyreactivity of antibodies to antigens (i.e., enhanced target antigen binding) and also the polyreactivity to unrelated proteins (i.e., non-specific binding to off-target proteins) [19,20,21,22]. Namely, there are two potential problems arising from low pH exposure: the loss of potency and the unexpected activities of antibodies.

### 2.3. Protein A Leaching and Toxicity

Harsh elution conditions such as the above can cause the leaching of Protein A. Protein A can leach during acid elution and contaminate the antibody fractions [7,8]. If the leached Protein A contaminates the eluted antibody samples, it will be strongly bound by the antibody. This causes difficulty in the dissociation of the antibody–Protein A complex and thereby the purification of the leached Protein A. The leached Protein A can be toxic when injected, as in leucocyte toxicity [23]. For example, an antibody–Protein A complex has been reported to cause a loss in leukocyte due to necrosis mechanism [23]. Leached Protein A has been linked to toxicity in animals and humans.

## 3. Elution of Proteins from Agarose Gel Electrophoresis

**Figure 1:**
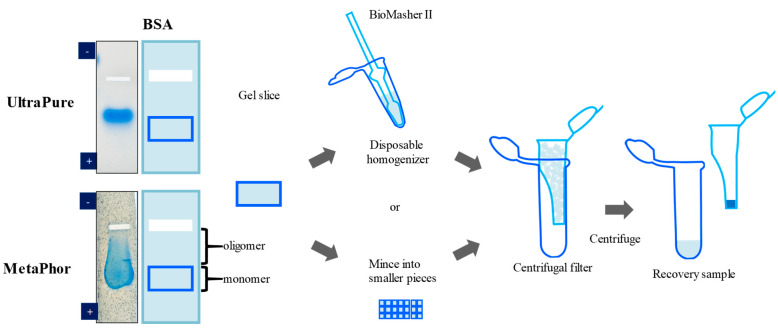
Schematic illustration of the extraction of proteins from agarose native gel electrophoresis. BSA was subjected to agarose native gel electrophoresis using 1% UltraPure agarose run at 100 V for 50 min or 5% MetaPhor agarose run at 100 V for 90 min. BSA migrates toward the anode side. After confirming the migration position of BSA via CBB staining, the agarose slice containing BSA (boxed) was excised from the unstained gel and used for extraction. Protein extraction from the gel slice was performed mechanically using a disposable homogenizer BioMasher II or manually. The crushed gel was immersed into the extraction buffer (0.1 M His/0.1 M MES) and centrifuged using Centrifugal Filter ATTOPREP MF: note that this commercial filter has a semi-permeable membrane attached to the bottom of the filter in angle to minimize filter clogging. The eluted fraction was analyzed via electrophoresis (see Figure 2).

**Figure 2:**
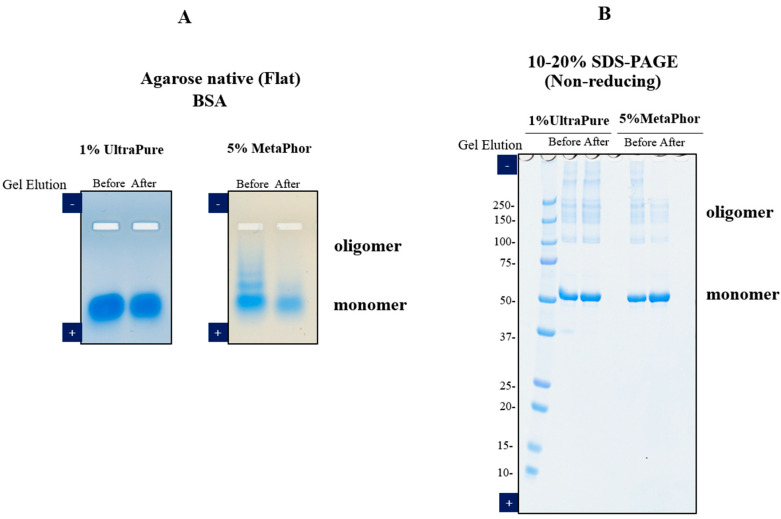
Electrophoresis of BSA before and after gel elution. BSA (2 μg/lane) before and after gel elution were analyzed using two different electrophoresis methods. First: (**A**) agarose native gel electrophoresis. Left panel: BSA sample before and after extraction from 1% UltraPure agarose. Electrophoresis was performed with 1% UltraPure agarose at 100 V for 50 min. Right panel: BSA sample before and after extraction from 5% MetaPhor agarose. Electrophoresis was performed with 5% MetaPhor agarose at 100 V for 90 min. (**B**) 10–20% SDS-PAGE under non-reducing conditions, BSA sample before and after extraction from 1% UltraPure agarose or 5% MetaPhor agarose. SDS-PAGE was conducted at 30 mA for 50 min.

We have developed a novel electrophoresis technique based on agarose gel and a His/MES (histidine/2-(*N*-morpholino)ethanesulfonic acid) buffer system at pH 6.1 [9,10,11]. Using this technique, we previously observed reasonable separation of recombinant antibodies from host HEK293 cell proteins, as only antibodies migrated toward the cathode [24]. While agarose gels, in particular those made using UltraPure agarose, are highly porous and therefore have the disadvantage of a weak size-sieving mechanism, this characteristic is advantageous for easy extraction [25]. Thus, we have attempted to extract proteins from the gel using a simple homogenization and centrifugation procedure as a convenient purification method. This process and equipment were schematically depicted in Figure 1 using bovine serum albumin (BSA) as a model protein. BSA was subjected to agarose native gel electrophoresis on standard UltraPure agarose (1%) and high-resolution MetaPhor agarose (5%) followed by Coomassie Brilliant Blue (CBB) staining. As shown in Figure 1 (left), the BSA migrated toward the anode in both gels, with smearing on the MetaPhor agarose. The observed difference between the UltraPure and MetaPhor gels is most likely due to the standard BSA containing monomers and some oligomers. High resolution MetaPhor—but not low resolution UltraPure—agarose can separate the monomers and oligomers. The bands were excised from the unstained gel (boxed in Figure 1), homogenized mechanically using BioMasher II or minced manually (Figure 1, middle) and spun in the centrifuge tube to collect the filtrate (Figure 1, right). The filtrate was then concentrated in the centrifuge tube using an angled semi-permeable membrane filter (Figure 1, right) and subjected to SDS-PAGE. Figure 2 shows the analysis of BSA before and after gel extraction. Agarose native gel electrophoresis of the extracted BSA from the UltraPure and MetaPhor agarose gel is shown in Figure 2A. Only one BSA band is seen on the 1% UltraPure gel (before extraction). Its extraction showed a single band after extraction, as expected. On the contrary, 5% MetaPhor agarose showed multiple bands (before extraction), from which only the monomer band was extracted. Thus, only the monomer band was observed after extraction, indicating that gel extraction of BSA can be used to purify the monomers, because 5% MetaPhor agarose gel can separate BSA species. This was confirmed using a SDS-PAGE analysis. As shown in Figure 2B, the band profile was identical before and after gel extraction using 1% UltraPure agarose due to its inability to separate oligomers, while the monomer was enriched after extraction from the 5% Metaphore agarose gel. This clearly demonstrates that protein can be readily purified using the above simple procedure, when the gel type and concentration are adequately adjusted.

**Figure 3:**
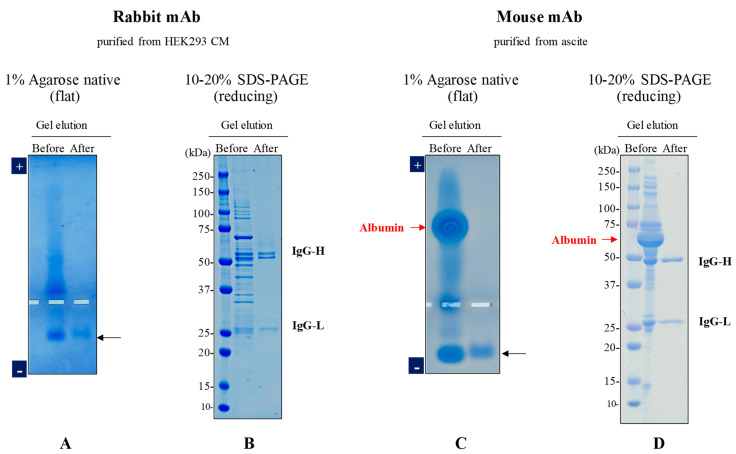
Electrophoresis of antibodies before and after electrophoresis and extraction. Agarose native gel electrophoresis (1% UltraPure agarose gel) was performed on the culture medium containing the rabbit monoclonal antibody expressed in HEK293 cells or the mouse ascites containing the mouse monoclonal antibody. The antibody migrated to the cathode side, while the impurities migrated to the anode side, leading to separation of the antibody. After confirming the migration position via CBB staining of a lane, an agarose slice containing the antibody was excised and used to extract the antibody. Antibodies (1–2 μg/lane) before and after extraction were analyzed using two different electrophoresis methods. (**A**) 1% UltraPure agarose native gel electrophoresis at 100 V for 50 min for rabbit antibody. (**B**) 10–20% SDS-PAGE under reducing conditions at 30 mA for 50 min for the rabbit antibody. (**C**) 1% UltraPure agarose native gel electrophoresis at 100 V for 60 min for the mouse antibody. (**D**) 10–20% SDS-PAGE under reducing conditions at 30 mA for 50 min for the mouse antibody.

Next, HEK293 cell cultures expressing a monoclonal antibody (mAb) were subjected to 1% UltraPure agarose native gel electrophoresis. As shown in Figure 3A (Before), the gel electrophoresis shows a band (namely a protein with an isoelectric point, pI, above 6.1) migrating toward the cathode corresponding to the antibody (shown by arrow) with the majority of contaminants migrating toward the anode. This band, corresponding to the antibody, was extracted as described above and analyzed via the same electrophoresis. As shown in Figure 3A (After), a single faint band was observed, perhaps due to insufficient extraction and concentration. An SDS-PAGE analysis confirmed the agarose gel analysis. As shown in Figure 3B, the SDS-PAGE showed multiple bands before extraction (Before) and primarily heavy and light chain bands after extraction (After). Similarly, a mouse monoclonal antibody was purified from ascites using agarose native gel electrophoresis followed by gel elution. As shown in Figure 3C (Before), the native electrophoresis shows a band corresponding to the antibody (indicated by arrow) migrating toward the cathode, while albumin migrated toward the anode. The SDS-PAGE analysis in Figure 3D also shows the intense BSA band of the ascites sample (Before) and only heavy and light chain bands of the highly purified antibody after extraction (After). Thus, separation via the current agarose native gel electrophoresis and extraction via homogenization were found to be effective in antibody purification.

## 4. Mixed-Mode Chromatography

**Figure 4:**
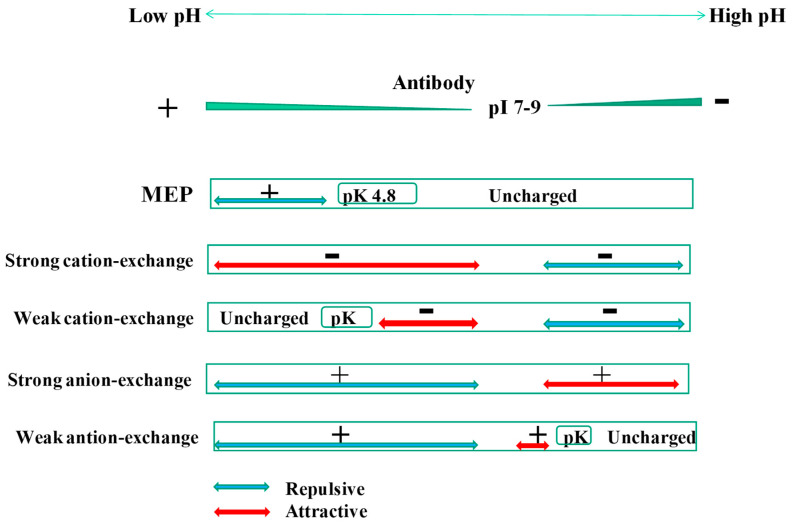
Charged states of several mixed-mode resins and antibodies as a function of pH.

**Figure 5:**
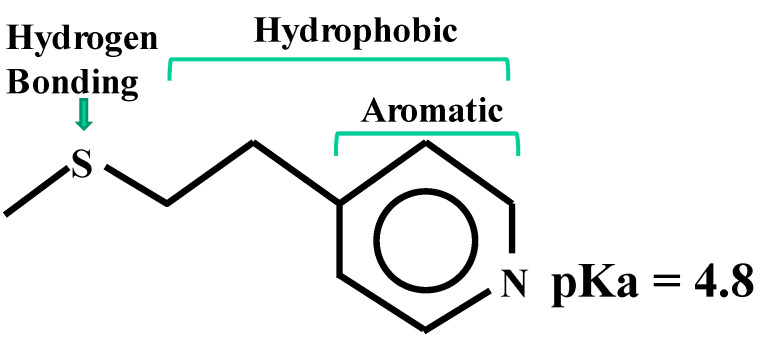
Structure of the MEP (4-Mercapto-ethyl-puridine) ligand.

As described earlier in the introduction, mixed-mode chromatography possesses a part of Protein A functionality, i.e., more than one binding mechanism. In relation to the charged state of antibodies, the charged state of several mixed-mode resins is schematically depicted in Figure 4. Antibodies in general have isoelectric points (pI) around 7–9, and hence increasing negative charges above this pH (blue triangle) and increasing positive charges below this pH (green triangle). The MEP (Figure 4, 2nd row), whose structure is shown in Figure 5, is positively charged roughly below pH 4.8, above which it is uncharged. Thus, there is no electrostatic interaction above pH 4.8 nor repulsive interaction with the antibody below this pH (shown by the blue arrow). The strong cation-exchange mixed-mode resin has a positive charge throughout the pH range, thus conferring an attractive interaction below the pI of antibodies (shown by the red arrow) and repulsive interaction above the pI. The weak cation-exchange mixed-mode resin is uncharged below its pK, above which it is negatively charged, indicating an attractive interaction between the pK of the resin and the pI of the antibodies (see Figure 4, 4th low). The strong anion-exchange mixed-mode resin is positively charged throughout the pH range, thereby conferring repulsive interaction below the pI of the antibodies and attractive interaction above the pI. Conversely, the weak anion-exchange mixed-mode resin is positively charged below its pK and uncharged above the pK. As depicted in Figure 4 (last row), this resin causes repulsive interaction below the pI of the antibodies (blue arrow) and attractive interaction between the pI of the antibodies and the pK of the resin. It is noted that all five of these mixed-mode resins have hydrophobic/aromatic binding mechanisms and/or hydrogen bonding function, which are independent of the pH. This in turn means that antibodies can bind to the resins hydrophobically and via hydrogen bonding and that rendering the resins uncharged may be insufficient to elute the antibodies. Rendering strong charge repulsion may overwhelm the hydrophobic/aromatic and hydrogen bonding interactions to cause antibody elution.

Mixed-mode chromatography has been primarily used as a polishing step following Protein A chromatography. There is a unique advantage of using mixed-mode chromatography after Protein A or other chromatography that results in the high ionic strength of the eluted protein solution. Many single-mode chromatography processes require adjustment of the pH, ionic strength and salt concentration. Because of their multiple binding mechanisms, mixed-mode resin can bind proteins under widely different solvent conditions. For example, high salt concentration suppresses the ionic binding mechanism, but enhances hydrophobic interactions. Low salt concentration may be insufficient for hydrophobic interactions but is favorable for ionic interactions. We focus on the application of mixed-mode chromatography to the capture step of antibody harvests.

### 4.1. MEP

MEP HyperCel resin (see Table 1 for vendors) was developed to mimic Protein A affinity columns, i.e., to capture antibodies and gain the low pH elution of bound antibodies [26,27,28]. This resin, shown in Figure 5, binds proteins at neutral pH through both hydrophobic–aromatic interactions and hydrogen bonding. As depicted in Figure 4, it is uncharged around a neutral pH, meaning that there is no electrostatic binding mechanism between the positively charged antibodies and MEP resin. Since protein binding is based on generic hydrogen and hydrophobic properties, any proteins can bind to this resin; there is no specificity to antibodies, unlike the strong specific antibody binding of Protein A. While carrying zero net charge at a neutral pH, this resin becomes positively charged with decreasing pH due to titration of the pyridine ring on the resin with a pK of 4.8. Since antibodies are positively charged at and below pH 4.8, they undergo charge repulsion from the positively charged MEP ligand. Since antibodies are more positively charged with decreasing pH, charge repulsion from the MEP is enhanced at a lower pH. It is thus designed to elute bound antibodies under a mildly acidic pH. Those basic and neutral proteins that can bind to the MEP resin and are also positively charged around pH 4.8, which would be eluted as well, leading to antibody fraction contaminated with co-eluted proteins. Those proteins that are acidic and hence still negatively charged or neutral at around pH 4.8 would not elute or could even bind more tightly to the resin. For example, mildly acidic serum albumins that have a pI around 4.6–5.5 may not be easily eluted, if they do bind to the MEP resin.

**Figure 6:**
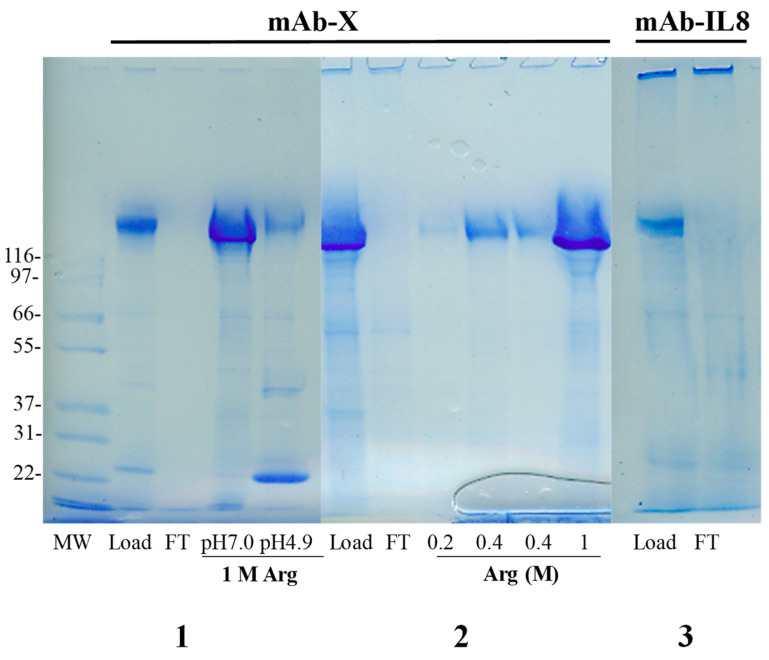
MEP chromatography of mAb-X and mAb-IL8. SDS-PAGE of chromatographic fractions. (**1**). mAb-X was loaded and eluted with 1 M arginine at pH 7.0 and pH 4.9. (**2**). mAb-X was loaded and eluted with arginine at 0.2, 0.4 and 1 M arginine at pH 7.0. (**3**). mAb-IL8 was loaded: only the load and FT are shown.

It has been shown with several antibodies and Fc-fusion proteins that they do bind to the MEP column when loaded directly from conditioned media expressing these proteins [29,30]. Examples of loading are shown in Figure 6 for two monoclonal antibodies (mAb-X developed against antigen X and mAb-IL8 against interleukin-8). As seen in the lane load (Figure 6), the mAb-X was fully retained by the column. Few proteins were found in the flow-through (FT), indicating that a majority of contaminating proteins were also retained. However, a large absorbance was observed in the flow-through fraction (chromatogram not shown). Nucleic acids and lipids may be in this flow-through fractions. In addition, CHO cell-conditioned media develop brownish color during cell culture, which also appeared to flow through the column. A similar result was obtained with conditioned medium expressing mAb-IL8 and Fc-fusion proteins. Since MEP can non-specifically bind contaminating proteins, it is critical to develop proper column washing conditions and also preferentially elute the bound antibodies that can separate them from more strongly bound contaminants.

The column was washed with 1 M NaCl, 10 mM phosphate, pH 7.0, which resulted in no elution of both mAb samples. This indicates that a high ionic strength alone cannot dissociate the bound mAb from the MEP resin and thus that the binding of mAb to MEP is not simply due to electrostatic interactions, consistent with no charges of the MEP resin. Next, the column was washed with 1 M arginine, 10 mM phosphate, pH 7.0. To our surprise, the bound mAb was efficiently eluted, as shown in Figure 6 (l M arginine pH 7.0) by an intense band on the gel. The column was then washed with 1 M arginine, 0.1 M citrate, pH 4.9, which resulted in little recovery of the mAb, but elution of contaminating proteins (see Figure 6). This shows preferential elution of the mAb at pH 7.0 with 1 M arginine without eluting many contaminants that eluted at pH 4.9. As 1 M NaCl could not elute the mAB, the observed elution via 1 M arginine suggests that this amino acid salt weakens hydrophobic interactions and possibly other interactions between the antibodies and MEP resin, which makes it effective in the elution of mAb from the MEP column. Such an effect of arginine is due to its favorable interaction with the proteins [31,32,33,34]. It is interesting to point out that the conductivity of 1 M arginine is much lower than that of the 1 M NaCl, although they are both monovalent [35]. Conductivity is not simply due to ionic strength, as different ionic species do exhibit different conductivity (e.g., even K ion vs. Na ion).

We have previously observed that arginine is not effective in Protein A chromatography above pH 5.0, even at 2 M [3,36]. On the contrary, 1 M arginine effectively eluted the bound mAb at pH 7.0 from the MEP columns, demonstrating that the MEP does not have binding affinity for antibodies that is comparable with Protein A. In addition, the majority of contaminating proteins normally flow through the Protein A column, in contrast to the MEP that can bind both mAb and contaminating proteins (see Figure 6, FT Lane). This indicates that the MEP is not as specific as Protein A for binding antibodies. Different arginine concentrations were tested. After loading conditioned media and washing with 10 mM phosphate, pH 7.0, the column was washed with 0.2 M arginine, pH 7.0, resulting in little elution of the mAb (Figure 6, Lane-0.2 M). Increasing the arginine concentration to 0.4 M resulted in more mAb elution (Figure 6, Lane-0.4 M), but still far below the level observed with 1 M arginine, suggesting that 0.4 M arginine may serve as an effective washing step.

Having established that arginine, which is an electrolyte, can elute mAb-X and mAb-IL8 as well as Fc-fusion proteins (not shown) from MEP HyperCel at a neutral pH, various electrolytes were tested for washing and elution. NaCl was totally ineffective as described above for both washing and elution at pH 7.0. A salting-in CaCl_2,_ which precipitates in phosphate, was tested in Tris–HCl buffer at pH 7.0 and showed little capacity—up to 1 M—to elute any proteins, including antibodies, although 0.15 M CaCl_2_ appears to remove some low molecular weight proteins. Another salting-in MgCl_2_ up to 1 M also showed no effects in 50 mM Tris-HCl, pH 7.0. Ammonium and sodium sulfate as well were not effective in eluting proteins.

**Figure 7:**
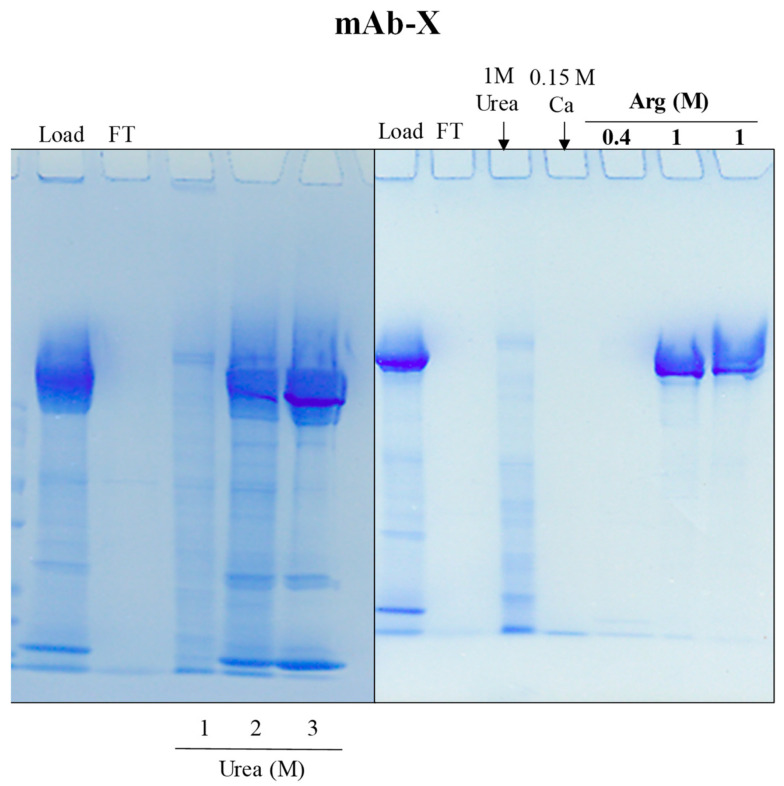
MEP chromatography of mAb-X. The bound mAb-X was eluted with 1, 2 and 3 M urea at pH 7.0. Purification of mAb-X from cell culture media was conducted with washes using 1 M urea, 0.15 M CaCl_2_ and 0.4 M arginine followed by elution with 1 M arginine twice, all at pH 7.0.

Next, the effectiveness of organic substances was examined on the elution of proteins from the MEP column. Ethanol wash at 5–20% was totally ineffective at eluting contaminants and both mAb samples. Urea at 1–3 M was tested: higher urea concentrations might affect the protein structure and stability. As shown in Figure 7, 1–3 M urea effectively eluted bound contaminating proteins, as shown by the bands on the gel. However, 2 and 3 M urea eluted the mAb as well, indicating that 1 M urea should be an effective washing solvent. The effect of urea appears to be selective at 1 M. Namely, it resulted in the elution of only contaminating proteins. Urea, as a polar compound, should be able to reduce both hydrophobic and polar interactions. In fact, urea has affinity for all of the amino acid side chains, whereas ethanol exhibits affinity only for non-polar side chains [37,38]. Urea slightly increases the dielectric constant of water [39] and hence should reduce polar interactions. In fact, urea forms more stable hydrogen bonds with peptide bonds than with water [37,38], thereby weakening hydrogen bonding between the protein and MEP resin. Thus, urea at high concentration (2–3 M) weakens hydrophobic and polar interactions to a sufficient extent that it causes the elution of the mAbs. Ehylene glycol (EG) at 50% was at least partially effective at eluting mAb-IL8. Lower concentrations of EG were found to be effective in washing the column. Glycerol, propanol and ethanol were not effective at eluting proteins, although propanol resulted in the removal of some contaminating pigments.

It appears from the observed effects of different washing solvents that low concentrations of arginine, urea and CaCl_2_ may be effective at removing contaminants. These washing steps were put together for the purification of mAb-X. Figure 7 (right panel) shows the SDS-PAGE of each step. There were no proteins in FT, as described above. The urea wash at 1 M resulted in the elution of many contaminating proteins with no apparent elution of the mAb. The CaCl_2_ wash at 0.15 M resulted in the elution of a low molecular weight band (lane 5). Additional contaminants were washed off using 0.4 M arginine. Finally, the bound mAb was eluted with 1 M arginine, pH 7.0.

**Figure 8:**
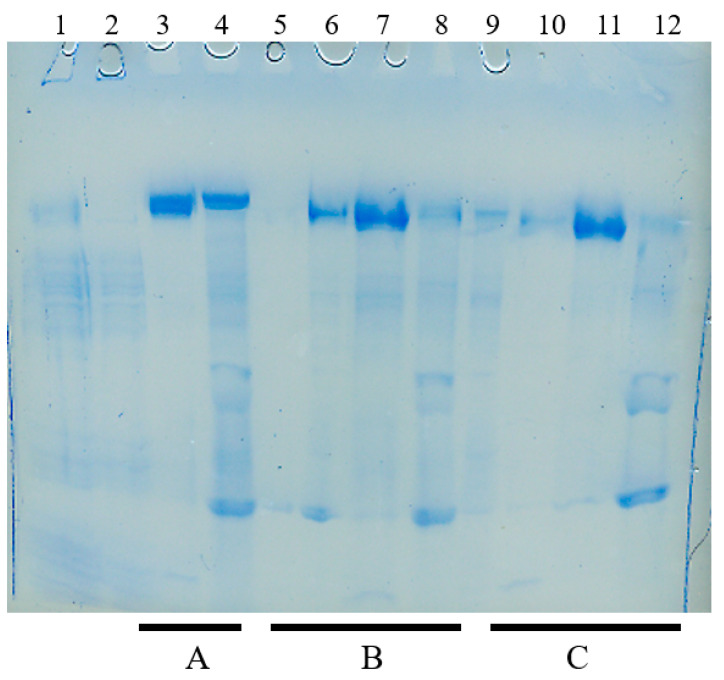
MEP chromatography of mAb-X. Three different protocols, A, B and C, were used as depicted in the inset to Figure 9. (**A**) Low pH elution (vendor’s protocol): no peak was observed with 100 mM acetate, 0.5 M NaCl. Lanes in SDS-PAGE: 1, Load; 2, FT; 3, 50 mM acetate (pH 5.0); 4, 0.1 M citrate (pH 3.0). (**B**) Arginine elution-1 (current protocol). Lanes in SDS-PAGE: 5, 25% 2-propanol/0.1 M NaCl (pH 7.0); 6, 35% ethylene glycol (pH 7.0); 7, 1 M arginine (pH 7.0); 8, 0.1 M citrate (pH 3.0). (**C**) Arginine elution-2 (previous protocol). Lanes in SDS-PAGE: 9, 1 M urea (pH 7.0); 10, 0.4 M arginine (pH 7.0), 11, 1 M arginine (pH 7.0); 12 0.1 M citrate (pH 3.0).

Next, the three protocols were compared on conditioned media containing mAb-X: protocol A, low pH elution; protocol B, propanol and EG; protocol C, urea and arginine for rinse. Figure 8 shows the results of the low pH wash and elution (lane 3, 4 and underlined A). No proteins eluted in the pH 5.5 rinse (100 mM acetate, 0.5 M NaCl). The pH 5.0 elution (50 mM acetate) resulted in 3.2 mg of mAb-X with reasonable purity (lane 3). Finally, the 0.1 M citrate, pH 3.0 wash resulted in the elution of many proteins, including some mAb-X (lane 4), indicating that the pH 5.0 elution is not sufficient for this particular antibody. Results from protocol B are shown in lanes 5–8 (underlined B). The rinse with 25% 2-propanol, 0.1 M NaCl, pH 7.0, resulted in the marginal elution of impurities (lane 5). The rinse with 35 % EG, pH 7.0, caused the elution of impurities as well as some mAb-X (lane 6). Elution with 1 M arginine, pH 7.0, resulted in 5 mg of mAb-X, a 1.5-fold higher recovery than that of the above pH 5.0 elution. Fewer proteins eluted in the pH 3.0 wash (lane 8). Results from protocol C are shown in lanes 9–12 (underlined C). Consecutive washes with 1 M urea (lane 9) and 0.4 M arginine (lane 10) resulted in the elution of 5 mg of highly purified mAb-X with 1 M arginine elution (lane 11). Some impurities were washed off using 0.1 M citrate, pH 3.0 (lane 12). The highest purity was achieved for the mAb-X using the above protocol. This is reasonable, as the previous protocol was developed using this antibody. It is thus essential to optimize the washing protocol for different antibodies.

**Figure 9:**
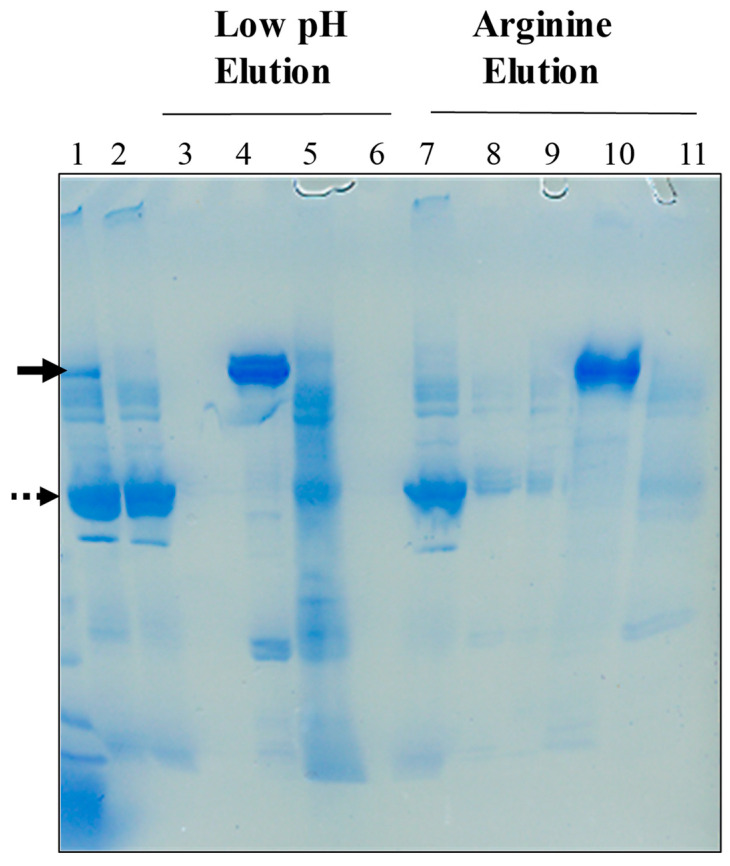
MEP chromatography of mAb-IL8 CM containing BSA. CM was spiked with BSA. Bound proteins were eluted with vendor’s protocol (lane 4 and 5) or the current protocol (lane 7–9). IL8-mAb is shown by a thick arrow and BSA by a dotted arrow. Lanes in SDS-PAGE: 1, Load; 2, FT; 3, 100 mM acetate/0.5 M NaCl, pH 5.5; 4, 50 mM acetate, pH 5.0; 5, 0.1 M citrate, pH 3.0 (vendor’s protocol). Arginine elution: lane 7, FT; 8, 25% 2-propanol/0.1 M NaCl, pH 7.0; 9, 35% EG, pH 7.0; 10, 1 M arginine, pH 7.0; 11, 0.1 M citrate, pH 3.0.

The ability of MEP chromatography to separate antibodies from BSA was tested using mAb-IL8 spiked with 50 mg BSA. Figure 9 shows the SDS-PAGE of various washing and elution conditions. The majority of BSA (dashed arrow) flowed through the column, as seen by the nearly equivalent intensity of the BSA band in the load (lane 1) and flow-through (lane 2). The 0.1 M acetate, 0.5 M NaCl, pH 5.5 wash resulted in no protein elution (lane 3), consistent with the above observation. Elution with 50 mM acetate, pH 5.0, effectively eluted the mAb (lane 4, see arrow), but with some contaminating bands. There are many bands eluting in the 0.1 M citrate, pH 3.0 wash (lane 5). The same experiment was repeated with different washes. The flow-through was similar to the above (lane 7), but more BSA proteins were eluted with 25% 2-propanol, 0.1 M NaCl, pH 7.0 (lane 8) and 35% EG, pH 7.0 (lane 9). These results suggest that a portion of BSA bound to the MEP via hydrophobic interactions, which can be weakened by these organic substances. The column was washed with 1 M arginine, pH 7.0, resulting in nearly a single IL8-mAb band (lane 10). There appears to be little protein in the final 0.1 M citrate, pH 3.0, consistent with extensive and effective washes via the intermediate washes. A comparison with the above low pH washes indicates that more effective washes with the organic substances were developed here.

MEP can also capture the single-chain variable domain (scFv) construct, which does not have the Fc-domain and hence does not bind to Protein A. HEK293 cell feedstock expressing a scFv showed a complete binding of the scFv under physiological conditions, resulting in greater than 80% recovery and purity upon pH 3.0 elution [40]. This scFv could not be eluted from a mixed-mode cation-exchange Capto MMC, while the mixed-mode anion-exchange Capto Adhere resulted in reasonable recovery and high purity from the MEP-eluted fractions [40]. The dynamic binding capacity of the MEP was determined using a monoclonal antibody from the bovine colostrum whey to be 10 mg/g resin at a flow rate of 1.6 cm/min independent of the loading protein concentration, and 18 mg/g resin at a 0.4 cm/min flow rate [41]. In another example of antibody purification from bovine milk, a large fraction of contaminating proteins flowed through the MEP column with complete binding of the antibody at a neutral pH, observed in variable scales. Combined with Capto MMC, MEP chromatography resulted in a highly purified antibody [42].

### 4.2. Hydrophobic-Ion Exchange Multimodal Chromatography

Mixed-mode resins that are different from MEP are composed of anion or cation exchange functions along with hydrophobic properties in a pH range around neutral; antibody binding occurs through both electrostatic and hydrophobic interactions, different from protein binding to the MEP, which occurs primarily through hydrophobic interactions. Since antibodies are mostly positively charged below pH 7–9, cation exchange mixed-mode resins are more useful than anion exchange mixed-mode resins. This is clearly shown in Figure 4, in which electrostatic binding can occur between cation-exchange and antibodies below pH 7–9 throughout the pH range for the strong exchange resins and above the pK for weak cation exchange resin. On the contrary, electrostatic binding between anion exchange resins can occur above pH 7–9 for the strong exchange resin and between pH 7–9 for the pK of the weak anion exchange resins. However, as with the MEP resin, hydrophobic interactions should be effective throughout the pH range. Elution of the bound antibodies is achieved via weakening hydrophobic interactions and electrostatic interactions or via increasing the charge repulsion to overwhelm the hydrophobic interactions. The latter becomes possible at a higher pH for the cation-exchange mixed-mode resins and at a lower pH for the anion exchange mixed-mode resins. Any electrolytes that can suppress both hydrophobic and electrostatic interactions should lead to antibody elution, even when the electrostatic binding mechanism is effective.

For example, cation-exchange mixed-mode resins include Eshmuno HCX and Capto MMC (Table 1 for vendors). Eshmuno HCX showed preferential binding of an antibody to the resin from CHO expression system at pH 6.0 in the presence of 100 mM NaCl [43]. In particular, a high binding capacity of the resin for the antibody on the order of 90 mg/mL was obtained under optimal conditions after chromatin extraction [43]. A combination of a hydrophobic ion-exchange mixed-mode, MEP, hydrophobic interaction and ion-exchange chromatography was compared with a typical Protein A-based platform purification of an antibody derived from CHO expression [13]. Some of these combinations, e.g., a combination of mixed-mode, MEP, cation exchange and anion exchange chromatography, resulted in a purity comparable to that of to the Protein A-based purification [13].

**Figure 10:**
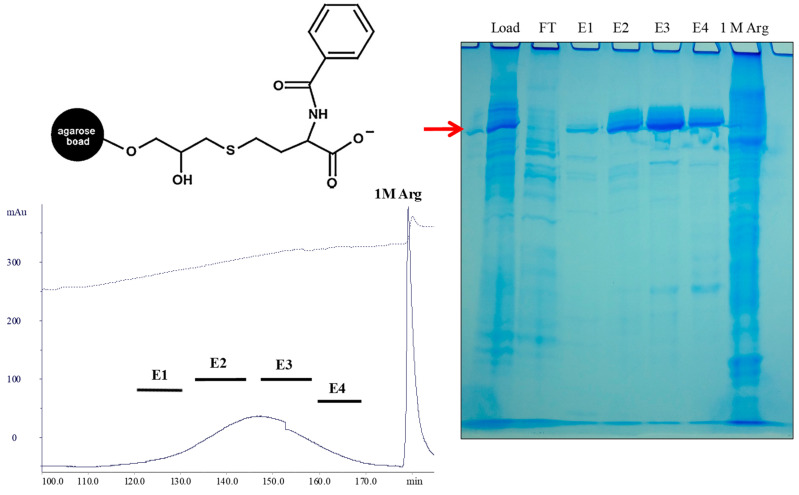
Purification of mouse monoclonal antibody using Capto MMC. Inset, structure of Capto MMC. Conditioned medium expressing a mouse antibody was loaded onto Capto MMC in PBS and eluted with a linear gradient from 0.3 M NaCl to 0.3 M arginine/0.3 M NaCl in 20 mM phosphate, pH 7.0. SDS-PAGE shows load, FT, eluted fractions of E1–E4 and final 1 M arginine (1 M) wash at pH 7.0. The arrow indicates the antibody band.

Availability of antibodies from different species plays a pivotal role in research and diagnostic applications. A cation-exchange mixed-mode resin, Capto MMC, has been used to capture mouse or rabbit antibody from cell harvest [44]. No difficulties were encountered in binding the mouse antibody to this resin. As shown in Figure 10, the mouse antibody (shown by arrow) bound to the Capto MMC column in both phosphate-buffered saline (PBS) and phosphate buffer, with some host cell proteins observed in the flow-through (FT). After evaluation of the elution conditions, a gradient elution from 0.3 M NaCl to 0.3 M arginine/0.3 M NaCl at pH 7.0 was used, resulting in the elution of the antibody in fractions E2–E4. The majority of contaminating proteins were eluted in the final 1 M arginine wash at pH 7.0. Highly purified mouse antibody was obtained by processing the Capto MMC pool to the hydroxyapatite chromatography.

**Figure 11:**
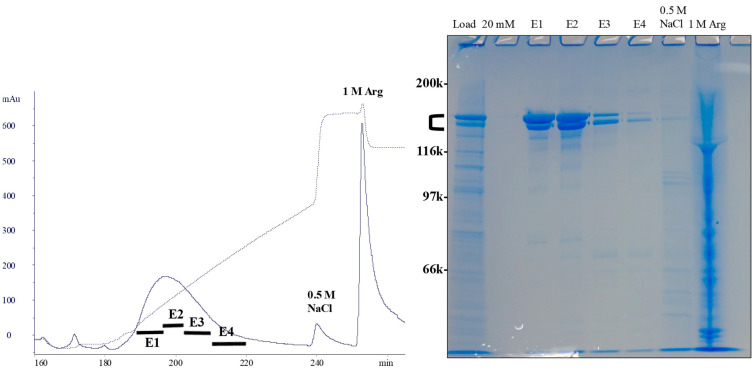
Purification of rabbit monoclonal antibody using Capto MMC mixed mode chromatography. Conditioned medium was adjusted to pH 6.0 and loaded onto Capto MMC at pH 5.2. After raising the column pH to 7.0, the bound proteins were eluted from 0 to 0.3 M NaCl. SDS-PAGE shows load, pH 7.0 wash (20 mM), eluted fractions of E1–E4 and final 0.5 and 1 M arginine wash (Arg).

This was not the case for rabbit antibody. It flowed through the Capto MMC column when directly loaded. Addition of 1 M ammonium sulfate promoted hydrophobic interactions, but still resulted in partial flow-through. Titration of the conditioned medium to pH 6.0, which increased the net positive charges on the rabbit antibody, conferred its complete binding to the resin that had been equilibrated at pH 5.2, where the Capto MMC is still negatively charged. The combination of these two changes enhanced electrostatic binding. Interestingly, when the column was washed with 20 mM phosphate, pH 7.0, the bound rabbit antibody was not eluted, meaning that once bound, there is sufficient binding strength. The bound antibody was eluted with a gradient of 0 to 0.3 M NaCl at pH 7.0. Figure 11 shows the elution profile and SDS-PAGE results. As shown in the gel, a doublet band of the rabbit antibody was observed due to heterogeneous glycosylation. It is clear that the bound antibody was observed in E1–E4 as a doublet. The final 1 M arginine wash resulted in the elution of contaminating proteins. No requirement of arginine for the elution of the rabbit antibody suggests that the hydrophobic contributions of its binding to Capto MMC are weaker than that occurring between this resin and human or mouse antibody. This in turn indicates the unique physical characteristic of the rabbit antibody.

**Figure 12:**
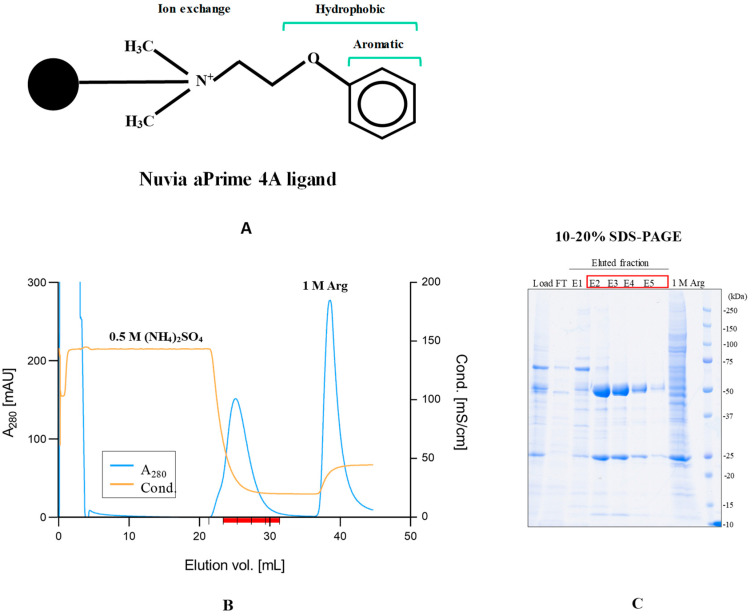
Purification of rabbit monoclonal antibody using Nuvia aPrime 4A mixed-mode chromatography. (**A**). Structure of Nuvia aPrime 4A ligand (**B**). Elution chromatogram of rabbit antibody. Conditioned medium was adjusted to pH 7.6 with Tris-buffered saline (TBS) and made 0.5 M ammonium sulfate and 1 M NaCl. The sample was loaded onto Nuvia aPrime and washed with 50 mM Tris-HCl at pH 7.6 containing 0.5 M ammonium sulfate and 1 M NaCl (20 CV). The bound proteins were eluted in one step with 100 mM MES, 150 mM NaCl (pH 7.0). (**C**) SDS-PAGE shows load, FT, eluted fractions of E1–E5 and final 1 M arginine wash. The red line in and red box in (**C**) indicate major fractions of antibody elution.

Nuvia aPrime 4A is a relatively new hydrophobic anion-exchange resin, as depicted in Figure 12A. Its user manual shows that antibodies can be bound in 20 mM sodium phosphate at pH 7.8 and purified via elution with 50 mM MES buffer at pH 6. First, we attempted this condition to purify a rabbit monoclonal antibody derived from a HEK293 cell culture harvest. However, this attempt failed due to no binding of the rabbit antibody, consistent with the above Capto MMC results that the rabbit antibody has weak hydrophobicity. When binding was attempted in the presence of 0.5 M ammonium sulfate that promotes hydrophobic interactions, the rabbit monoclonal antibody was able to bind to the Nuvia aPrime 4A resin at pH 7.6, again supporting the notion that this antibody species has weak hydrophobicity and requires a hydrophobic binding enhancer (i.e., ammonium sulfate). The column was then washed with 50 mM Tris-HCl (pH 7.6) containing 0.5 M ammonium sulfate and 1 M NaCl (20 CV), resulting in no elution of the bound rabbit mAb. The bound proteins were eluted with 100 mM MES and 150 mM NaCl at pH 7.0. As shown in Figure 12B, the majority of the rabbit antibody was eluted as peak fractions during the descending ammonium concentration. Many contaminant proteins were eluted with the final 1 M arginine wash (see sharp peak in Figure 12B). The SDS-PAGE analysis of eluted fractions in Figure 12C shows the elution of the rabbit antibody in E2–E4 and many contaminants in the 1 M arginine wash. The rabbit monoclonal mAb was eluted from the Nuvia aPrime 4A at pH 7 with an overall 90% recovery. It was shown that the rabbit monoclonal antibodies can be purified under mild conditions of pH 7–8 using mixed mode chromatography after modifying the binding conditions due to the weak hydrophobicity of rabbit antibodies.

### 4.3. Hydroxyapatite Chromatography

Hydroxyapatite (Table 1 for vendors) is composed of calcium phosphate and is not a typical mixed-mode column, as it does not have a hydrophobic contribution. It has two binding mechanisms. The calcium moiety provides chelation with carboxyl groups on proteins and also anionic exchange function, in which carboxyl chelation can be disrupted by phosphate competition. The phosphate moiety provides the cation-exchange function [45,46]. Although antibodies can be captured when loaded directly from cell culture supernatant, the phosphate buffer can weaken antibody chelating binding to hydroxyapatite [47]. Hydroxyapatite may offer a better alternative to Protein A for immunoglobulin A and immunoglobulin M, which both suffer from poor recovery during Protein A chromatography [48]. Hydroxyapatite has been shown effective as a polishing step to remove aggregated antibodies [49].

### 4.4. Salting-Out Chromatography

Salting-out chromatography traditionally refers to hydrophobic interaction chromatography (HIC), where a strong salting-out salt, such as ammonium sulfate, is routinely used. Lee et al. [50] developed an entirely different salting-out chromatography, termed “steric exclusion chromatography”, that uses polyethylene glycol (PEG) to salt-out large proteins (for example, immunoglobulin M, IgM) and viruses. PEG cannot be used in conventional HIC, as its hydrophobic property competes with protein binding to the HIC resin. Namely, this polymer has affinity for HIC resin, thereby suppressing protein binding to the resin. Instead, Lee et al. [50] used a hydroxy-substituted polymethacrylate monoliths (OH-monolith), which has a highly polar surface. Regardless of the binding mechanism, PEG causes salting-out of large molecules, as in crystallization, leaving low molecular species in the solution. In addition, as seen in protein crystallization, it enhances the accumulation of one particular solute species over other solutes on the surface of the monolith surface, leading to preferential accumulation of the target solute.

**Figure 13:**
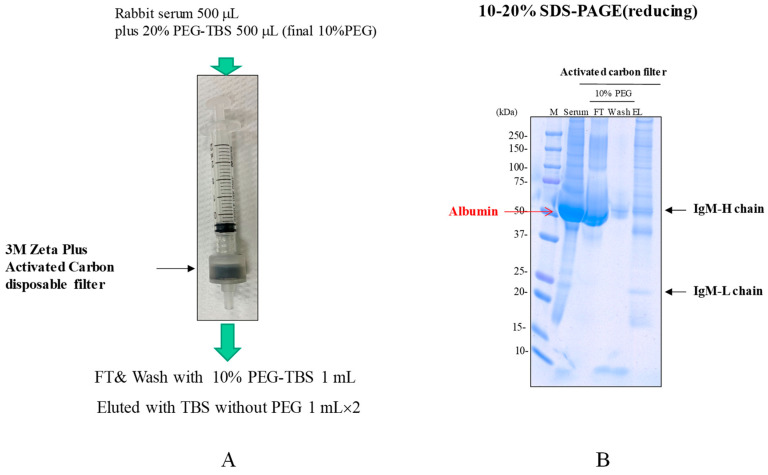
Purification of rabbit serum IgM using activated carbon filter and PEG 6000. Rabbit serum containing IgM was made 10% PEG and injected into the carbon filter. The bound proteins were eluted with TBS lacking the PEG. (**A**) Illustration of the activated carbon filter and scheme of purification methods. (**B**) SDS-PAGE shows load, FT, wash with the TBS containing 10% PEG and eluted fractions by TBS without PEG.

Inspired by the method of purifying IgM antibodies using the OH-monolith and PEG as described above, we have attempted to bind a rabbit antibody, which has shown difficulty in binding to mixed-mode resins, onto microporous activated carbon containing OH (and COOH) groups on its surface [51] and in the presence of PEG. It should be noted that the activated carbon is also hydrophobic, hence providing multiple binding mechanisms, including hydrophobic and ionic interactions and hydrogen bonding [52]. PEG could suppress the hydrophobic binding. Activated carbon is currently used industrially for the removal of impurities, including endotoxins, nucleic acids, host cell proteins and pigment components in biopharmaceutical media [53,54]. We attempted to purify IgM from rabbit serum. The rabbit serum was mixed at 1:1 with Tris-buffered saline (TBS) at pH 7.6 containing 20% PEG 6000 (final concentration of 10% PEG) in a lab-scale 3M Zeta plus activated carbon filter (Table 1 for vendors) and was injected into the filter using a disposable syringe (Figure 13A). The bound proteins were eluted in step with the TBS lacking the PEG. The load, FT, wash and eluted fractions were analyzed using SDS-PAGE (Figure 13B). As seen in the SDS-PAGE profile, many serum proteins, including serum albumin (see red arrow) and immunoglobulin G, were removed in the flow-through (FT). In addition, IgM was not eluted, even after washing with 10% PEG solution (wash). Bound IgM was eluted with TBS (pH 7.6) without PEG (EL). The purity of the IgM antibodies was still low in this one-step purification method from serum, but this principle shows a possibility of purifying IgM antibodies with high purity by using a PEG gradient elution, and also that it can be combined with other clarification and purification procedures.

## 5. Conclusions

We have summarized in this review various purification techniques, i.e., the non-affinity purification of antibodies that do not depend on Protein A chromatography. For research scale, agarose native gel electrophoresis was used to separate the antibodies from contaminants followed by their elution from the gel. For non-affinity chromatographic purification, we described mixed-mode chromatography, including MEP, ion-exchange mixed-mode chromatography, hydroxyapatite chromatography and salting-out chromatography. Advantages of using these non-affinity technologies, e.g., no adverse effects of high-affinity Protein A chromatography, mild elution conditions and cost saving, are described, as summarized in Table 1, where merits and demerits as well as brief remarks for each non-affinity purification of antibodies are given. As a final remark, it would be of great interest to compare antibodies prepared using the low pH elution from Protein A chromatography and non-affinity purification in relation to structural diversity that potentially causes the polyreactivity of antibodies [19,20,21,22,55].

## Figures and Tables

**Table 1:** Merits and demerits of non-affinity antibody purification.

Method	Merit	Demerit	Remark	Providers
Gel elution	No binding to solid support.	Small scale Recovery.	Separation dependent on pI of Ab.	Ultrapure agarose (Thermo Fisher Scientific) Metaphor agarose (Lonza)
MEP	Direct loading from harvest Simple elution.	Low DBC. No Ab specificity.	Critical column wash.	MEP HyperCel (Sartorius, etc.)
Ion-exchange mixed-mode	Direct loading from harvest Robust binding.	Difficult elution.	Avoid harsh elution condition.	CaptoMMC (Cytiva) Nuvia aPrime 4A (Bio-Rad Laboratories)
Hydroxyapatite	Binding with high salt or arginine.	Unpredictable binding and elution.	Choice of pH above neutral. Elution by NaCl followed by phosphate.	Hydroxyapatite (Bio-Rad Laboratories)
Salting-out	High DBC.	Requirement of salting-out condition.	Differential elution of contaminants and Ab.	3M Zeta Plus Activated Carbon disposable filter (3M)

DBC, dynamic binding capacity (binding capacity during loading and dependent on flow rate of loading). Ab, antibody. The merits and demerits for Protein A are described in the text in great detail.

## Data Availability

No new data were created or analyzed in this study. Data sharing is not applicable to this article.
